# Hydrogel-Based Therapeutic Strategies for Post-Cholecystectomy NAFLD: Targeting Bile Acid Signaling, Gut Microbiota, Inflammation, and Hepatic Fibrosis

**DOI:** 10.3390/gels12020179

**Published:** 2026-02-20

**Authors:** Georgiana-Andreea Marinescu, Alexandra-Daniela Rotaru-Zavaleanu, Emil-Tiberius Trasca, Elena-Irina Caluianu, Oana Taisescu, Andrei Gresita, Madalina Iuliana Musat, Dumitru Radulescu, Razvan Mercut, Citto-Iulian Taisescu

**Affiliations:** 1 Department of Surgery, “Dr. Ștefan Odobleja” Military Emergency Clinical Hospital, 200749 Craiova, Romania; marinescu_georgiana.andreea@yahoo.com (G.-A.M.); etrasca@yahoo.com (E.-T.T.); irina.caluianu@umfcv.ro (E.-I.C.); dr_radulescu_dumitru@yahoo.com (D.R.); 2 Doctoral School, University of Medicine and Pharmacy of Craiova, 200349 Craiova, Romania; 3 Department of Epidemiology, University of Medicine and Pharmacy of Craiova, 2–4 Petru Rares Street, 200349 Craiova, Romania; alexandra.rotaru@umfcv.ro; 4 General Surgery Department, University of Medicine and Pharmacy of Craiova, 200349 Craiova, Romania; 5 Department of Human Anatomy, University of Medicine and Pharmacy of Craiova, 200349 Craiova, Romania; 6 Department of Physiology, University of Medicine and Pharmacy of Craiova, 200349 Craiova, Romania; citto.taisescu@umfcv.ro; 7 Department of Scientific Research Methodology, University of Medicine and Pharmacy of Craiova, 2–4 Petru Rares Street, 200349 Craiova, Romania; madalina.musat@umfcv.ro; 8 Department of Plastic and Reconstructive Surgery, University of Medicine and Pharmacy of Craiova, 200349 Craiova, Romania; mercut.razvan@umfcv.ro

**Keywords:** post-cholecystectomy NAFLD, gut–liver axis, bile acid modulation, microbiota-targeted therapy

## Abstract

Post-cholecystectomy non-alcoholic fatty liver disease (NAFLD), now encompassed within metabolic dysfunction-associated steatotic liver disease (MASLD), is increasingly linked to persistent disruption of bile acid kinetics and gut–liver axis signaling after gallbladder removal. Continuous bile delivery to the intestine reshapes the bile acid pool, perturbs FXR–FGF19/TGR5 pathways, remodels gut microbiota, and compromises epithelial barrier integrity, collectively promoting portal endotoxemia, chronic hepatic inflammation, and fibrogenic remodeling. Hydrogel-based biomaterials offer a mechanistically aligned therapeutic platform for this setting because they enable localized, sustained, and stimuli-responsive interventions at intestinal or hepatic sites. Functional hydrogels can sequester excess bile acids, protect and deliver probiotics/prebiotics/postbiotics, reinforce mucosal barrier function, and provide controlled release of anti-inflammatory or antifibrotic agents with reduced systemic exposure. In this review, we map emerging hydrogel strategies relevant to post-cholecystectomy NAFLD across four pathogenic nodes, bile acid dysregulation, dysbiosis, inflammation, and fibrosis, and highlight design principles (polymer chemistry, charge/hydrophobicity balance, mucoadhesion, and pH/redox/enzyme responsiveness) that enable targeted modulation of the gut–liver axis. Finally, we identify key translational gaps, including the lack of post-cholecystectomy-specific experimental models and standardized outcome measures integrating bile acid profiling, microbiome readouts, and hepatic histology. Hydrogel technologies represent a promising route toward localized and multimodal therapy in metabolic liver disease, warranting focused preclinical validation and clinical development.

## 1. Post-Cholecystectomy NAFLD: Clinical and Biological Context

### 1.1. Clinical Relevance of NAFLD After Cholecystectomy

Non-alcoholic fatty liver disease (NAFLD), recently reframed under the concept of metabolic dysfunction-associated steatotic liver disease (MASLD), is currently the most prevalent chronic liver disease worldwide and a major hepatic manifestation of systemic metabolic dysfunction [1]. Increasing evidence suggests that cholecystectomy, one of the most frequently performed abdominal surgical procedures, is associated with a higher risk of NAFLD development and progression [2].

Observational studies, population-based cohorts, and meta-analyses consistently report a higher prevalence of NAFLD/MASLD among individuals who have undergone cholecystectomy compared with non-cholecystectomized controls [3]. Importantly, this association often persists after adjustment for traditional metabolic risk factors, including obesity, type 2 diabetes mellitus, and dyslipidemia, indicating that gallbladder removal may represent an independent contributor to hepatic steatosis [4]. Post-cholecystectomy patients frequently exhibit adverse metabolic alterations, including increased insulin resistance, dysregulated lipid metabolism, impaired bile acid signaling, and a higher prevalence of metabolic syndrome. These findings reinforce the conceptualization of NAFLD as a multisystem metabolic disorder, in which the liver is particularly susceptible to disturbances in enterohepatic circulation, inflammatory signaling, and metabolic homeostasis. Within this framework, cholecystectomy may act as a metabolic modifier that accelerates hepatic fat accumulation and promotes disease progression in metabolically or genetically predisposed individuals [5,6].

### 1.2. Pathophysiological Alterations Induced by Cholecystectomy Relevant to NAFLD

The removal of the gallbladder profoundly alters bile acid homeostasis and disrupts the tightly regulated gut–liver axis, creating a biological environment conducive to NAFLD development and progression [7].

In the absence of the gallbladder, bile secretion into the intestine becomes continuous and uncoupled from food intake [8]. This persistent luminal exposure to bile acids affects not only lipid digestion but also the signaling functions of bile acids, which act as key metabolic regulators [9]. Cholecystectomy is associated with qualitative and quantitative changes in the bile acid pool, including alterations in the balance between primary and secondary bile acids. These shifts have important downstream effects on metabolic regulation [10]. Bile acids serve as ligands for nuclear and membrane receptors such as farnesoid X receptor (FXR) and Takeda G protein-coupled receptor 5 (TGR5), which play central roles in hepatic lipid metabolism, glucose homeostasis, and energy expenditure [11,12]. Post-cholecystectomy dysregulation of FXR–FGF19 and TGR5 signaling has been linked to increased hepatic lipogenesis, impaired fatty acid oxidation, and disturbances in systemic glucose metabolism, all of which favor hepatic steatosis [13].

Simultaneously, chronic alterations in intestinal bile acid exposure reshape the gut microbial ecosystem. Cholecystectomy-associated intestinal dysbiosis affects bacterial species involved in bile acid biotransformation and short-chain fatty acid production, further amplifying metabolic imbalance [14]. These microbial alterations are often accompanied by impairment of the intestinal epithelial barrier, leading to increased intestinal permeability [15]. Consequently, bacterial-derived products such as lipopolysaccharides can translocate into the portal circulation, resulting in low-grade endotoxemia that amplifies systemic inflammation and immune dysregulation, with potential for distant organ involvement, including neuroinflammatory complications in severe clinical contexts [16,17].

This sustained inflammatory input from the gut activates hepatic immune pathways, promotes hepatocellular injury, and stimulates hepatic stellate cells, thereby contributing to chronic liver inflammation and the gradual development of fibrosis. Collectively, these mechanisms illustrate how cholecystectomy generates a distinct intestinal and hepatic microenvironment, characterized by altered bile acid signaling, dysbiosis, barrier dysfunction, and inflammation [18].

Taken together, these changes highlight a pathophysiological setting in which localized, targeted, and mechanistically driven therapeutic interventions, particularly at the level of the intestine and gut–liver axis, are especially relevant, providing a strong biological rationale for exploring hydrogel-based strategies in post-cholecystectomy NAFLD [19,20].

## 2. Materials and Methods

This narrative review was conducted using a structured literature search to identify relevant studies on hydrogel-based therapeutic strategies for post-cholecystectomy NAFLD and modulation of the gut–liver axis. Searches were performed in PubMed, Web of Science, and Scopus for English-language articles published between January 2000 and January 2026. Search terms included combinations of “cholecystectomy,” “NAFLD,” “MASLD,” “gut–liver axis,” “bile acids,” “gut microbiota,” “inflammation,” “fibrosis,” “hydrogel,” “biomaterials,” “drug delivery,” “bile acid sequestration,” “mucoadhesive,” and “stimuli-responsive hydrogel.” Reference lists of selected articles were manually screened to identify additional relevant studies. Original research articles and high-quality reviews relevant to intestinal or hepatic targeting, bile acid modulation, microbiota interaction, inflammation control, and antifibrotic strategies were included. Studies unrelated to gut or liver applications were excluded.

## 3. Hydrogels as a Therapeutic Platform in Post-Cholecystectomy NAFLD

Hydrogels are three-dimensional, crosslinked polymeric networks capable of retaining large amounts of water while maintaining structural integrity. Owing to their tunable physicochemical properties and high biocompatibility, hydrogels have emerged as versatile platforms for biomedical applications, including drug delivery and tissue-targeted therapies [21,22,23,24,25,26,27,28]. In the context of post-cholecystectomy NAFLD, hydrogels offer several advantages over conventional pharmacological approaches.

A key strength of hydrogel-based systems lies in their ability to enable localized drug delivery. Depending on their composition and responsiveness, hydrogels can be engineered to target specific anatomical sites relevant to NAFLD pathogenesis, such as the ileum and colon, critical regions for bile acid signaling and microbiota modulation, or the liver itself through injectable or implantable formulations [29,30]. This spatial precision is particularly valuable in post-cholecystectomy patients, in whom pathological processes are strongly driven by alterations at the level of the gut–liver axis [20].

**Figure 1:**
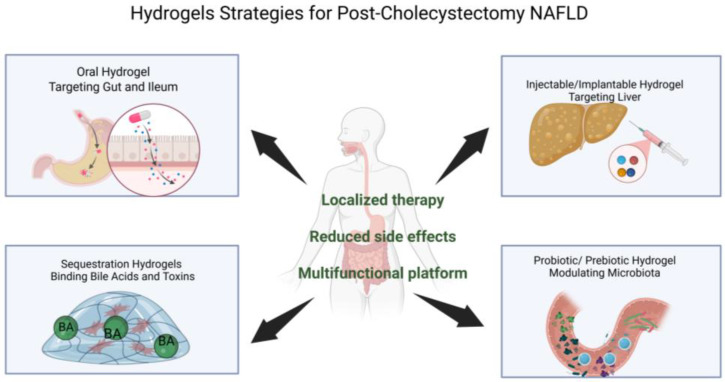
Hydrogels Strategies for Post-Cholecystectomy NAFLD (Created in BioRender. https://BioRender.com/073bwbg, (accessed on 15 January 2026)).

Another major advantage is the capacity for controlled and stimuli-responsive release. Hydrogels can be designed to respond to environmental cues such as pH gradients, enzymatic activity, or microbial metabolites, allowing drug release to occur selectively within the intestinal tract or hepatic microenvironment [31]. This controlled release profile improves therapeutic efficacy while minimizing fluctuations in drug concentration that are commonly observed with conventional oral or systemic therapies [32] (Figure 1).

By confining therapeutic activity to targeted sites, hydrogel-based approaches also contribute to a reduction in systemic adverse effects, a critical consideration in the long-term management of chronic metabolic liver diseases [33]. This is particularly relevant for agents acting on bile acid pathways, inflammatory cascades, or fibrogenic signaling, which may otherwise exert off-target effects when administered systemically [34].

Importantly, hydrogels offer a therapeutic modality that extends beyond classical drug delivery. Certain hydrogel systems are capable of sequestration functions, physically binding luminal molecules such as bile acids or bacterial-derived toxins [35]. This property enables modulation of pathogenic drivers at their source, rather than solely relying on receptor-mediated pharmacological inhibition. In the post-cholecystectomy setting, where excessive or dysregulated intestinal bile acid exposure plays a central role, such sequestration-based strategies are especially attractive [36]. Collectively, these features position hydrogels as a flexible and multifunctional therapeutic platform, well suited to address the complex, multi-layered pathophysiology of post-cholecystectomy NAFLD and to support targeted interventions along the gut–liver axis.

Comparative advantages over conventional therapies

To establish the translational rationale for hydrogel-based interventions in post-cholecystectomy NAFLD, their performance must be compared against conventional therapeutic approaches across four key domains.

The first one is bile acid sequestration. Traditional sequestrants (cholestyramine, colesevelam) bind bile acids non-selectively throughout the gastrointestinal tract, causing poor tolerability (gastrointestinal side effects in 30–50% of patients), fat-soluble vitamin malabsorption, and premature binding in the proximal intestine [37]. Hydrogel-based sequestrants overcome these limitations through pH- and enzyme-responsive site-specific delivery to the terminal ileum and colon, enabling selective binding of pathogenic bile acids while preserving physiological FXR signaling proximally. Functional group engineering provides tunable binding selectivity unattainable with conventional resins, while stimuli-responsive activation minimizes upper gastrointestinal side effects and reduces dosing frequency.

Regarding the probiotic delivery, we must mention that conventional probiotic formulations suffer from poor gastric survival (<10^3^ CFU/g reaching the colon), rapid luminal clearance (2–6 h), and vulnerability to bile acid toxicity which is a challenge amplified post-cholecystectomy [38]. Hydrogel encapsulation within alginate-chitosan or pectin-based matrices maintains viability > 10^6^ CFU/g (>1000-fold improvement), prolongs intestinal retention through mucoadhesion, shields bacteria from bile acid toxicity, and enables co-delivery of prebiotics for integrated synbiotic strategies [39].

Anti-inflammatory therapy using systemic agents (NSAIDs, corticosteroids) risk hepatotoxicity and fails to achieve therapeutic concentrations at the gut–liver interface. Hydrogel-based delivery achieves 10- to 50-fold higher mucosal drug concentrations with minimal systemic absorption. Mucoadhesive systems provide dual functionality by combining pharmacological delivery with physical barrier protection of inflamed epithelium, while stimuli-responsive release triggered by ROS or acidic pH ensures site-selective drug activation [40].

Also, systemic antifibrotic agents, such as pirfenidone, demonstrate poor hepatic accumulation (<5% reaching the liver) and dose-limiting toxicity. Injectable hydrogel platforms enable direct intrahepatic delivery with sustained release over weeks, and can simultaneously deliver multiple modalities (small molecules, siRNA, growth factors, cellular therapeutics), while their tunable mechanical properties independently modulate hepatic stellate cell behavior [41].

The post-cholecystectomy setting particularly favors hydrogel-based approaches because continuous bile acid exposure, bile acid-driven dysbiosis, regional intestinal inflammation, and barrier dysfunction operate simultaneously, requiring multifunctional platforms capable of integrating bile acid sequestration, probiotic protection, anti-inflammatory delivery, and epithelial barrier reinforcement within spatially and temporally controlled systems. This multifunctionality represents the core translational advantage of hydrogel-based interventions over conventional single-target therapies.

## 4. Hydrogel-Based Bile Acid Sequestration Strategies

### 4.1. Bile Acids as a Key Therapeutic Target After Cholecystectomy

Bile acids play a central role in the pathogenesis of NAFLD following cholecystectomy, acting not only as detergents involved in lipid digestion but also as potent metabolic signaling molecules. After gallbladder removal, bile secretion into the intestine becomes continuous and no longer synchronized with food intake, resulting in increased and prolonged intestinal exposure to bile acids. Post-cholecystectomy bile acid dysregulation manifests through quantifiable alterations in bile acid pool composition and enterohepatic cycling: clinical studies demonstrate a 2–3-fold increase in circulating total bile acids, with primary bile acids (cholic acid and chenodeoxycholic acid) elevated by 40–60% and the conjugated/unconjugated ratio increasing from approximately 3:1 to 5–7:1 [42]. This continuous bile acid flux, rather than meal-synchronized bolus delivery, fundamentally alters downstream signaling cascades and profoundly affects enterohepatic signaling and metabolic regulation.

Persistent luminal bile acid flow contributes to aberrant activation of bile acid-responsive receptors, particularly farnesoid X receptor (FXR) and Takeda G protein-coupled receptor 5 (TGR5). The FXR–FGF19 axis shows characteristic post-cholecystectomy dysfunction: while basal FGF19 levels may remain preserved due to continuous ileal bile acid exposure, postprandial FGF19 peaks are reduced by 30–50% compared to individuals with intact gallbladders [43]. This loss of pulsatile FGF19 signaling impairs hepatic lipid metabolism regulation and bile acid synthesis feedback, resulting in enhanced hepatic de novo lipogenesis, impaired lipid oxidation, and disturbances in glucose homeostasis. Similarly, TGR5 pathway activation demonstrates altered temporal dynamics, with diminished meal-synchronized GLP-1 secretion despite maintained or elevated basal pathway activity, further influencing energy expenditure and inflammatory pathways [44]. Together, these changes promote hepatic fat accumulation and metabolic dysfunction.

These bile acid alterations directly reshape intestinal microbiome composition through selective pressure on bile-tolerant taxa. The continuous bile acid exposure favors Gram-negative Bacteroidetes, particularly bile acid-metabolizing genera such as *Bacteroides* and *Alistipes*, often shifting the Firmicutes/Bacteroidetes ratio [45]. Functionally critical taxa harboring bile salt hydrolase (BSH) and 7α-dehydroxylase activities, primarily *Clostridium* cluster XIVa, *Eubacterium*, and *Ruminococcus* species, demonstrate variable responses depending on substrate availability and intestinal transit time [46]. This microbial functional shift reduces secondary bile acid production (particularly deoxycholic acid and lithocholic acid), further dysregulating FXR/TGR5 signaling and creating a feed-forward cycle promoting metabolic dysfunction [47].

Moreover, excessive or unbalanced bile acid signaling can exacerbate hepatic inflammation both directly and indirectly through effects on the gut microbiota and intestinal barrier integrity. In this context, bile acids emerge as a nodal pathogenic driver linking intestinal alterations to hepatic steatosis, inflammation, and disease progression in post-cholecystectomy NAFLD. Targeting bile acids at the intestinal level therefore represents a biologically sound and mechanistically focused therapeutic strategy.

### 4.2. Hydrogels as Bile Acid Sequestration Platforms

Bile acid sequestration is a well-established therapeutic strategy for reducing plasma cholesterol and indirectly modulating hepatic metabolism. Traditional agents such as cholestyramine act by inhibiting the enterohepatic circulation of BA; however, their effectiveness is limited by poor tolerability, non-specific binding, and gastrointestinal side effects (bloating, constipation, malabsorption) [48]. These limitations have stimulated the development of novel, functionalized polymeric platforms with enhanced binding specificity and improved biocompatibility.

Isothermal titration calorimetry (ITC) measurements from functionalized chitosan hydrogels report binding affinities for bile acids in the range of K = 10^4^–10^6^ M^−1^, with enthalpy-driven interactions predominating for conjugated bile acids and entropy-driven contributions for unconjugated species [49]. Quaternary ammonium-functionalized dextran hydrogels demonstrate sequestration capacities exceeding 80% for sodium cholate under physiologically relevant conditions, while pectin-based systems crosslinked with calcium ions achieve 65–75% binding efficiency for deoxycholic acid, with selectivity tunable through carboxylate density [50]. Recent chitosan-graft-polyacrylic acid systems report maximum bile acid adsorption capacities of 180–240 mg/g dry polymer, representing a 3–4-fold improvement over conventional cholestyramine resin under equivalent conditions [51].

Within this framework, hydrogels have emerged as promising systems for the selective sequestration of BA within the intestinal lumen [52]. These three-dimensional networks, capable of retaining large amounts of water, can be engineered with cationic (e.g., primary or quaternary amines) or hydrophobic (e.g., alkyl or aromatic) functional groups that mediate electrostatic and host–guest interactions with the amphiphilic structure of bile acids [53]. This dual binding mechanism enables both electrostatic attraction to the bile acid’s carboxyl group and hydrophobic interactions with the rigid steroidal core, thereby enhancing binding affinity and functional specificity [54].

As reviewed by Stanciu et al. (2023) [55] in Gels, hydrogels used for BA sequestration can be classified based on their polymer source into two main categories: natural gels (e.g., chitosan, alginate, pectin, cellulose) and synthetic gels (e.g., polyacrylate, poly(vinyl alcohol), poly(2-hydroxyethyl methacrylate)). Each class offers specific advantages: natural hydrogels are biodegradable and compatible with gut microbiota, whereas synthetic variants allow fine-tuning of network properties such as crosslink density, swelling capacity, and porosity. Their chemical versatility also enables the inclusion of selective recognition elements for specific BA species such as chenodeoxycholic vs. deoxycholic acid, which is particularly important in pathological contexts such as post-cholecystectomy, where qualitative shifts in bile acid composition critically affect hepatic homeostasis [55].

A major conceptual advantage of hydrogel-based systems over classical sequestrants lies in their capacity for localized action along defined intestinal segments [56]. By adjusting their swelling and degradation properties, these materials can be activated in response to intestinal pH or specific stimuli (enzymes, microbiota), minimizing systemic absorption and adverse effects [57]. Furthermore, rather than drastically depleting BA, hydrogels can selectively reshape the luminal bile acid pool, preserving signaling through FXR and TGR5 receptors. This allows for a more physiologically balanced approach that may reduce systemic inflammation, improve hepatic lipid handling, and restore homeostasis in complex conditions such as non-alcoholic fatty liver disease (NAFLD) [58] (Table 1).

Cationic hydrogels (bearing primary or quaternary ammonium groups) are preferred for broad-spectrum bile acid sequestration due to electrostatic attraction to the carboxylate/sulfonate groups present on conjugated bile acids, which constitute the dominant fraction post-cholecystectomy (conjugated–unconjugated ratio 5–7:1) [59]. Anionic hydrogels, while less efficient for direct electrostatic capture, offer complementary utility: their pH-responsive swelling behavior enables selective retention in defined intestinal segments, and their interaction with the positively charged mucin layer facilitates mucoadhesion and prolonged epithelial contact, relevant for modulating ileal FXR activation by controlling the local bile acid microenvironment rather than achieving wholesale depletion [60]. A hybrid zwitterionic design therefore represents the most mechanistically rational approach for simultaneously sequestering pathogenic bile acid species while preserving the residual FXR/FGF19 signaling necessary for metabolic homeostasis.

From a structural and mechanical standpoint, properties such as porosity, crosslinking degree, hydration capacity, and molecular release kinetics are key determinants of sequestration efficiency [61]. As shown by Stanciu et al. in their study, cationic dextran-based hydrogels functionalized with a high density of N-alkyl-N,N-dimethylammonium chloride moieties exhibit a strong capacity to bind bile acids, highlighting the relevance of polymer charge and amphiphilicity in bile acid sequestration. By evaluating sodium cholate adsorption under equilibrium conditions in both aqueous and physiologically relevant ionic environments, the authors demonstrated that amphiphilic hydrogel architectures containing ammonium groups of differing polarity achieved superior sorption performance. Adsorption behavior across all polymer formulations conformed well to established isotherm models, including Langmuir, Dubinin–Raduskevich, and Temkin, supporting a predominantly surface-driven, energetically heterogeneous binding process. Notably, these dextran-based hydrogels displayed exceptionally high bile acid binding capacities, reaching values exceeding 1 g of sodium cholate per gram of polymer, underscoring their potential as efficient bile acid–targeting materials in therapeutic and metabolic applications [62].

As shown in both in vitro and in vivo studies, certain modified chitosan-based hydrogels incorporating fatty acids or quaternary ammonium groups can achieve sequestration efficiencies comparable to or surpassing cholestyramine, while offering superior biocompatibility.

Crosslinking density exerts opposing effects on sequestration capacity and responsiveness to inflammatory stimuli. Lightly crosslinked networks (<5 mol%) exhibit high swelling ratios (Q = 20–50 g/g) and rapid bile acid uptake but show uncontrolled release under the elevated ROS and acidic pH (5.5–6.5) characteristic of inflamed mucosa. Conversely, highly crosslinked matrices (>15 mol%) maintain structural integrity under inflammatory conditions but demonstrate reduced swelling and slower bile acid diffusion, limiting sequestration efficiency. Intermediate crosslinking densities (8–12 mol%), particularly in dual-network systems combining covalent and ionic crosslinks, provide the optimal balance: maintaining sequestration capacity > 75% while enabling stimuli-responsive retention in inflamed intestinal segments. Incorporation of ROS-cleavable crosslinkers (e.g., thioketal or boronate ester bonds) represents an emerging strategy whereby oxidative stress at sites of active inflammation triggers network degradation and localized payload release, a design principle particularly relevant to the dysbiotic, pro-inflammatory environment of post-cholecystectomy intestinal mucosa [63].

Hydrogels represent a new generation of intelligent platforms for bile acid sequestration, capable of combining molecular selectivity, localized action, and an optimal biocompatibility profile. Future research should focus on stimuli-responsive hydrogels with controlled release and self-regulating capacity, aiming not only to optimize therapeutic efficacy but also to integrate these materials into personalized treatment strategies for dyslipidemia and metabolic liver disorders.

## 5. Hydrogels for Modulation of the Gut Microbiota

### 5.1. Post-Cholecystectomy Dysbiosis and Its Contribution to NAFLD

Cholecystectomy-induced alterations in bile acid flow and composition exert a profound impact on the gut microbial ecosystem, positioning intestinal dysbiosis as a central mediator in the development and progression of post-cholecystectomy NAFLD [64]. Bile acids are key determinants of microbial selection within the gut, and their continuous luminal presence after gallbladder removal reshapes microbial composition and function [65]. Clinical and experimental studies indicate a reduction in short-chain fatty acid (SCFA)–producing bacteria, which play a protective role in maintaining intestinal barrier integrity, regulating inflammation, and supporting metabolic homeostasis [66]. Concurrently, there is often an expansion of pro-inflammatory bacterial taxa, contributing to increased intestinal and systemic inflammatory tone. These microbial shifts are closely linked to metabolic disturbances characteristic of NAFLD, including insulin resistance and hepatic lipid accumulation [67].

Moreover, dysbiosis following cholecystectomy affects the microbial conversion of primary to secondary bile acids, a process essential for maintaining balanced bile acid signaling [14]. Disruption of this conversion alters the bile acid pool and further perturbs FXR and TGR5 pathways, creating a feed-forward loop between bile acid dysregulation, microbial imbalance, and hepatic metabolic dysfunction. Through these interconnected mechanisms, post-cholecystectomy dysbiosis amplifies gut–liver axis signaling and contributes to the progression from simple steatosis to inflammatory and fibrotic liver disease [68].

### 5.2. Hydrogels as Platforms for Microbiota-Targeted Therapy

The gut microbiota is increasingly recognized as a critical mediator in post-cholecystectomy non-alcoholic fatty liver disease (NAFLD), modulating bile acid composition, gut barrier integrity, and hepatic inflammation. As such, targeted microbiome restoration has emerged as a rational and promising therapeutic approach. Hydrogel-based systems offer distinct advantages in this context, enabling localized, protective, and sustained delivery of microbiota-modulating agents throughout the intestinal tract [69].

A wide range of hydrogel systems has been explored as carriers for probiotics, prebiotics, and postbiotics, thanks to their tunable swelling behavior, biocompatibility, and responsiveness to environmental cues. Encapsulation of probiotic strains within hydrogel matrices protects them from gastric acid and bile toxicity, significantly improving their viability and metabolic function upon reaching the colon. This is especially relevant for post-cholecystectomy patients, whose altered bile acid dynamics may otherwise reduce probiotic survival [70]. Alginate-chitosan core–shell systems maintain probiotic viability at >10^6^ CFU/g through simulated gastric and intestinal conditions, representing a >1000-fold improvement over non-encapsulated formulations. Extrusion-based encapsulation preserves >85% viability for Lactobacillus rhamnosus GG, outperforming spray-drying (40–60% survival) due to reduced thermal and osmotic stress during processing [71].

Beyond serving as delivery vehicles for conventional therapeutics, hydrogel-based systems offer direct mechanisms for microbiota modulation that are particularly relevant to post-cholecystectomy NAFLD. Probiotic encapsulation and protection represents a primary strategy: hydrogel matrices shield viable probiotic strains (*Lactobacillus*, *Bifidobacterium*, bile acid-metabolizing species) from gastric acid and bile toxicity during gastrointestinal transit, ensuring their viability upon reaching the colon [72]. *Lactobacillus rhamnosus* GG and *L. plantarum* WCFS1 are preferentially indicated for epithelial barrier repair via upregulation of tight junction proteins (occludin, claudin-3, ZO-1), while *Bifidobacterium longum* demonstrates superior efficacy in bile acid biotransformation and FXR modulation [73]. *Akkermansia muciniphila* shows particular relevance in post-cholecystectomy dysbiosis through mucin layer restoration and TGR5-mediated GLP-1 secretion enhancement [74]. Studies demonstrate that alginate-chitosan microbeads and pectin-based hydrogels maintain probiotic viability above 10^6^ CFU/g through simulated gastric and intestinal conditions, compared to <10^3^ CFU/g for non-encapsulated strains [75]. Following colonic delivery, encapsulated probiotics demonstrate superior colonization and metabolic activity, including enhanced production of short-chain fatty acids (SCFAs) and secondary bile acid biotransformation. Prebiotic substrate delivery through hydrogel carriers provides complementary microbiota modulation: inulin, fructo-oligosaccharides (FOS), and galacto-oligosaccharides (GOS) embedded within pH- or enzyme-responsive matrices are released specifically in the colon, where they selectively enrich beneficial taxa [76]. In vivo studies in dysbiotic rodent models show that prebiotic-loaded hydrogels increase *Lactobacillus* and *Bifidobacterium* abundance by 2–3 log units while reducing pathogenic *Enterobacteriaceae* and *Clostridium* species, restoring microbial diversity indices toward control values [77]. Importantly, sustained prebiotic release from hydrogels produces more stable microbiota shifts compared to bolus administration, as gradual substrate availability prevents overgrowth of opportunistic fermenters. Postbiotics, particularly butyrate (physiological range 2–8 mM), exert direct anti-inflammatory effects through HDAC inhibition and GPR41/GPR43 activation independently of microbial viability, offering advantages in stability and reproducibility. Hydrogel-encapsulated butyrate achieves 3–5-fold higher colonic mucosal concentrations with sustained release over 24–48 h compared to free administration. However, live probiotics retain superiority in adaptive microbiome remodeling and in situ production of a broader metabolite spectrum [78].

In antibiotic-induced dysbiotic murine models, pectin-zein hydrogels delivering *L. plantarum* restore Firmicutes/Bacteroidetes ratios within 7–14 days, with sustained colonization confirmed at day 21. Injectable hydrogels co-delivering *B. longum* and inulin reduce hepatic TNF-α and IL-6 by 40–55% alongside restoration of BSH-active microbial populations in NAFLD models, providing direct evidence for gut-liver axis remodeling [79].

For instance, pH-responsive hydrogels offer a promising strategy for colon-targeted delivery of curcumin (CUR). Zhang et al. developed a system in which CUR-loaded carboxymethyl chitosan (CC) microspheres were embedded in a hyaluronic acid–gelatin (HA–GE) hydrogel network, formed via amide bond crosslinking. The swelling behavior of the hydrogel was dependent on the HA-to-GE ratio, with higher GE reducing and higher HA increasing expansion capacity. The hydrogel remained stable in acidic gastric conditions (pH 1.2), preventing premature CUR release. Under intestinal pH (6.8–7.4), the structure swelled and degraded, enabling sustained and site-specific CUR release in the colon. This system prolonged drug availability and significantly improved localized therapeutic efficacy in IBD models [80].

Moreover, Xu et al. designed a thermosensitive, mucoadhesive hydrogel (TMNR) for targeted IBD therapy. Formed from Pluronic F127, HA, and α-cyclodextrin, the hydrogel transitioned from liquid at 4 °C to gel at 37 °C. The addition of α-CD enhanced stability, maintaining structure up to six days. Incorporation of a melanin–gallium complex enabled anti-inflammatory action. In vivo studies confirmed strong intestinal adhesion, mechanical resilience under peristalsis, and prolonged retention in colitis models. TMNR demonstrated effective colonic targeting, barrier function, and extended therapeutic duration [81].

Pectin-based hydrogels enable colon-targeted drug delivery due to their resistance to gastric and small intestinal conditions and degradability by colonic enzymes. pH- and enzyme-responsive systems combining pectin and polyacrylamide (PAM) have been developed to deliver budesonide specifically to the colon, exhibiting pH-dependent swelling and biphasic release. For quercetin (QT), whose oral bioavailability is limited, Jing et al. designed a colon-specific delivery system (COS–CaP–QT) using pectin/Ca^2+^ microspheres crosslinked with low molecular weight oligo-chitosan (COS). Compared to high molecular weight chitosan, COS offers superior film-forming, anti-swelling, and penetrative properties, enhancing microsphere stability and colon-specific release via enzymatic and microbial degradation [82]. In a recent study, Zhang et al. developed a redox-responsive hydrogel using thiol-functionalized hyaluronic acid (HASH) for inflammation-specific targeting in colitis. Varying thiolation levels (20–60%) yielded HASH variants, with HASH60% showing the fastest ROS-triggered gelation in the presence of H_2_O_2_. Adhesion assays confirmed enhanced mucosal binding via disulfide bonding with colonic tissue thiols, especially under oxidative conditions. In vivo imaging in DSS-induced colitis mice revealed strong, selective accumulation of HASH60% in inflamed colons, while no hydrogel formed in healthy controls, highlighting the hydrogel’s ROS-dependent, site-specific activation and adhesion at inflamed intestinal sites [83].

Importantly, the anti-inflammatory and barrier-protective effects of these hydrogel systems produce indirect microbiota modulation by reshaping the intestinal luminal environment. Reduction in mucosal inflammation decreases oxygen diffusion into the colonic lumen, favoring anaerobic beneficial commensals over facultative anaerobic pathogens such as *Enterobacteriaceae*. Reinforcement of epithelial barrier integrity limits translocation of bacterial products and reduces inflammatory tone, creating a more favorable niche for SCFA-producing *Faecalibacterium* and *Roseburia* species. Studies using mucoadhesive hydrogels delivering anti-inflammatory agents demonstrate not only reduced colonic cytokine levels but also restoration of microbial composition, with increased alpha diversity and recovery of bile acid-metabolizing bacterial functions [84]. This bidirectional relationship, wherein hydrogel-mediated inflammation control supports beneficial microbiota, which in turn reinforces barrier function and suppresses inflammation, illustrates the multifaceted mechanisms through which these platforms modulate the gut–liver axis in post-cholecystectomy NAFLD.

Importantly, hydrogel systems offer the advantage of gradual, adaptive release, unlike conventional bolus-dose interventions. The dynamic interplay between the hydrogel network and the gut environment, via stimuli such as pH shifts, enzymatic activity, or redox conditions, allows for responsive, long-term modulation of microbial communities. This adaptability is particularly valuable in the context of post-cholecystectomy NAFLD, where microbial composition and bile acid signaling evolve over time (Table 2).

Collectively, hydrogel-based platforms for microbiota-targeted therapy operate through direct mechanisms, including probiotic protection and colonization enhancement, prebiotic substrate delivery for selective bacterial enrichment, and incorporation of antimicrobial agents targeting pathogenic species, as well as indirect mechanisms whereby anti-inflammatory and barrier-protective effects reshape the luminal environment to favor beneficial commensals. The ability to combine these approaches within responsive, mucoadhesive delivery systems provides a multifaceted strategy for restoring gut microbiota homeostasis in post-cholecystectomy NAFLD. Unlike systemic antibiotics or poorly targeted probiotic supplements, hydrogel platforms enable spatially and temporally controlled microbiota modulation aligned with disease-specific dysbiotic patterns, including expansion of bile-tolerant pathobionts and loss of secondary bile acid–producing species characteristic of cholecystectomy-associated gut–liver axis dysfunction.

## 6. Hydrogel-Based Anti-Inflammatory Strategies

### 6.1. Intestinal and Hepatic Inflammation in Post-Cholecystectomy NAFLD

Chronic low-grade inflammation represents a central pathogenic mechanism in post-cholecystectomy NAFLD, arising at the interface between the intestine and the liver and progressively extending to systemic metabolic dysfunction. Following cholecystectomy, alterations in bile acid flow and gut microbiota composition compromise intestinal barrier integrity, facilitating the translocation of bacterial-derived products into the portal circulation [85].

One of the most extensively described mechanisms is endotoxemia driven by lipopolysaccharides (LPS), which originate from Gram-negative bacteria and gain access to the liver through increased intestinal permeability. Elevated portal LPS levels activate innate immune pathways within the liver, including Kupffer cells and hepatic stellate cells, triggering the release of pro-inflammatory cytokines and chemokines. This process reinforces hepatic insulin resistance and promotes hepatocellular lipid accumulation [86].

The resulting activation of the gut–liver axis sustains a state of chronic, low-grade inflammation that is characteristic of NAFLD progression. Rather than manifesting as acute inflammatory injury, this persistent inflammatory milieu exerts cumulative effects over time, driving the transition from simple steatosis to steatohepatitis and predisposing to fibrotic remodeling [87]. In post-cholecystectomy patients, continuous bile acid exposure and dysbiosis act synergistically to maintain this inflammatory tone, highlighting inflammation as a key therapeutic target.

### 6.2. Hydrogels as Anti-Inflammatory Therapeutic Platforms

Chronic low-grade inflammation along the gut–liver axis is a central pathological feature in post-cholecystectomy NAFLD, perpetuated by mucosal injury, dysbiosis, and increased translocation of microbial products. In this context, hydrogel-based systems offer a versatile and targeted approach to dampening intestinal and hepatic inflammation by combining localized drug delivery, barrier reinforcement, and responsive bioactivity [14]. Hydrogels have been widely investigated as carriers for anti-inflammatory agents, antioxidants, and epithelial-protective molecules, enabling their sustained release directly at inflamed intestinal sites. This strategy minimizes systemic exposure while concentrating therapeutic action at the mucosal interface [88]. Beyond pharmacological delivery, mucoadhesive hydrogel systems can function as protective physical barriers. These formulations adhere to the intestinal epithelium and shield it from bile acids, bacterial toxins, and inflammatory stimuli, a key advantage in post-cholecystectomy contexts where bile acid-induced epithelial injury is amplified [89].

Huang et al. designed an anti-inflammatory hydrogel platform using sulfated alginates (Algs) with high chemokine-binding capacity, combined with PB nanozymes for potent antioxidant activity. These composite hydrogels demonstrate multifunctionality by integrating adhesion, chemokine sequestration, and oxidative stress modulation, demonstrating significant reduction in pro-inflammatory cytokine expression (TNF-α reduced by 65%, IL-1β by 58%, IL-6 by 62% compared to untreated controls) and enhanced ROS scavenging capacity [90]. Similarly, Yang et al. developed a collagen–hyaluronic acid hydrogel enhanced with catechol-functionalized gallic acid, conferring strong wet adhesion and antioxidant properties. Incorporation of Prussian blue nanoparticles (PBNPs) as nanozymes enabled efficient ROS scavenging [91].

Further advancing hydrogel applications, Zhipeng Zhou’s group created a dual-network biomaterial combining methacrylated gelatin (GelMA) with snail mucus-derived glycosaminoglycan (AFG), the latter serving as a potent anti-inflammatory agent. This hydrogel effectively trapped pro-inflammatory cytokines and suppressed their expression via inhibition of the NF-κB signaling cascade. It also facilitated macrophage polarization toward an M2 anti-inflammatory phenotype. In diabetic wound models, the AFG/GelMA hydrogel reduced TNF-α expression by 3.2-fold and IL-6 by 2.8-fold compared to controls, while increasing M2 macrophage markers (CD206, Arg-1) by approximately 2-fold [92].

The physical properties of a hydrogel’s adhesive environment seem to influence macrophage inflammatory behavior through the regulation of the transcriptional coactivator YAP. Specifically, macrophages adhering to softer hydrogel substrates exhibited a subdued inflammatory response compared to those on stiffer materials, an effect linked to reduced YAP expression and diminished nuclear localization [93]. In another study, Xu et al. proposed a chitosan–catechol-based mucoadhesive gel designed for rectal administration of sulfasalazine. This formulation enhanced drug delivery efficiency and safety, offering a valuable alternative to oral administration in managing ulcerative colitis [94].

Similarly, Xinyue Ge and colleagues developed a multifunctional hydrogel for localized ulcerative colitis therapy. Upon application, it adhered firmly to ulcerated mucosa and provided sustained dexamethasone (DEX) release, which acted on the TLR4–NF-κB axis to shift macrophages from a pro-inflammatory (M1) to anti-inflammatory (M2) state, achieving 70% reduction in disease activity index (DAI) scores and significant suppression of inflammatory mediators (TNF-α, IL-1β, iNOS) with corresponding increase in anti-inflammatory markers (IL-10, Arg-1) [95].

Recent innovations in wet-adhesive hydrogels, especially those incorporating antioxidants like catechols, polyphenols, and Prussian blue nanoparticles, have shown strong efficacy in neutralizing reactive oxygen species (ROS). Materials embedded with polydopamine or quercetin exhibit enhanced ROS-scavenging ability [96]. Hydrogels composed of collagen or hyaluronic acid not only possess antioxidant activity but also adhere effectively to mucosal surfaces. By reducing oxidative stress, downregulating pro-inflammatory pathways like NF-κB, and promoting M2 macrophage polarization, these multifunctional hydrogels offer a promising therapeutic platform for inflammatory and oxidative disorders in the gastrointestinal tract [97]. The multifunctionality of hydrogel platforms, combining drug release, physical protection, and biochemical modulation, enables a layered anti-inflammatory effect that aligns well with the complex pathophysiology of NAFLD. Notably, these systems can be designed for spatial and temporal precision, responding to local pH, oxidative stress, or microbial enzymes to release their payloads only when and where needed [98]. Hydrogels represent a next-generation strategy for controlling intestinal inflammation in post-cholecystectomy NAFLD. By integrating localized anti-inflammatory delivery with epithelial protection and immune modulation, these platforms offer a sophisticated, site-specific method to restore intestinal homeostasis and indirectly attenuate hepatic immune activation [99].

Quantitatively, these hydrogel-based anti-inflammatory strategies demonstrate substantial efficacy in preclinical models. Cytokine suppression typically show significant reduction in TNF-α, IL-1β, and IL-6 levels compared to untreated controls [100]. ROS scavenging capacity, measured by DPPH or ABTS assays, shows 60–85% free radical neutralization. Macrophage polarization studies consistently demonstrate 2- to 3-fold increases in M2/M1 ratios, with corresponding increases in anti-inflammatory markers (IL-10, Arg-1, CD206) and decreases in pro-inflammatory markers (iNOS, TNF-α, IL-12). Disease activity indices in inflammatory bowel disease models show 50–80% improvement, while histological inflammation scores decrease by 40–65%. These quantitative benchmarks establish the translational potential of hydrogel platforms for managing gut–liver axis inflammation in post-cholecystectomy NAFLD [93].

## 7. Hydrogels and Hepatic Fibrosis

### 7.1. Hepatic Fibrosis as the Critical Determinant of Disease Progression

Hepatic fibrosis represents the pivotal pathological process that determines long-term outcomes in NAFLD, marking the transition from benign steatosis to progressive liver disease. While simple steatosis may remain clinically silent for extended periods, progression to steatohepatitis (NASH/MASH) and subsequent fibrotic remodeling is strongly associated with increased risks of cirrhosis, hepatocellular carcinoma, and liver-related mortality [101].

In post-cholecystectomy NAFLD, persistent metabolic stress, bile acid dysregulation, dysbiosis, and chronic low-grade inflammation converge to promote activation of hepatic stellate cells, the principal drivers of extracellular matrix deposition and fibrosis. Once initiated, fibrogenic pathways tend to become self-sustaining, rendering fibrosis a major therapeutic challenge [102]. Despite extensive research, effective antifibrotic therapies remain limited, and most current interventions primarily target upstream metabolic or inflammatory processes. Consequently, there is a critical unmet need for therapeutic strategies capable of directly modulating fibrogenic signaling and supporting tissue remodeling. In this context, fibrosis emerges as the key endpoint that ultimately dictates prognosis and therapeutic success in NAFLD, including in patients following cholecystectomy [103].

### 7.2. Hydrogel-Based Antifibrotic Interventions

Hydrogel-based systems have gained increasing attention as platforms for localized and targeted antifibrotic therapies, particularly in the liver, where spatial control of drug delivery is essential to maximize efficacy and minimize systemic toxicity. Injectable hepatic hydrogels can be engineered to conform to the liver microenvironment, enabling sustained release of therapeutic agents directly at sites of fibrotic activity [104]. These systems have been explored for the local delivery of antifibrotic compounds, including small molecules, biologics, and nucleic acid–based therapeutics such as siRNA. By concentrating therapeutic payloads within fibrotic regions, hydrogel-based approaches enhance target engagement while reducing off-target effects that have limited the clinical translation of many antifibrotic agents (Table 3) [105]. Curcumin-loaded hydrogels reduce hepatic TGF-β1 expression by 45–60% and α-SMA-positive cell density by 35–50% in NASH rodent models, with concurrent reduction in collagen deposition (Sirius Red quantification: 40–55% vs. controls). In CCl_4_-induced fibrosis models, injectable hydrogels delivering quercetin or salvianolic acid B reduce hepatic hydroxyproline content by 40–60% and downregulate TGF-β1/Smad3 signaling after 4-week administration, achieving 2–3-fold higher intrahepatic drug concentrations compared to systemic delivery at equivalent doses [106].

Bolinas et al. developed a hydrogel-based delivery system for mesenchymal stem cells (MSCs), which were intraportally administered to cirrhotic rats. Their formulation ensured high cell viability (89.0 ± 3.0%) and enabled a sustained MSC release over a two-week period. In vivo, the animals treated with the MSC-loaded hydrogel exhibited significantly improved liver regeneration markers, including increased liver volumes (FLR ratio: 0.57 ± 0.32) and weights (FLR ratio: 0.84 ± 0.05). Enzymatic analyses also indicated hepatoprotective effects, with reductions in AST (72.75 ± 14.17 U/L), ALT (46.00 ± 2.94 U/L), and ALP (135.67 ± 41.20 U/L) levels. Histological evaluation showed that fibrosis was notably reduced (4.52 ± 0.22%) in the hydrogel-treated group. Furthermore, cell retention within hepatic tissue was markedly higher when MSCs were delivered via hydrogel (37.30 ± 16.10 MSCs/mm^2^), compared to injection of MSCs alone (21.70 ± 22.10 MSCs/mm^2^). These findings support the hydrogel’s role in enhancing MSC therapeutic efficacy through improved localization, viability, and anti-fibrotic action [107].

Gu et al. designed a hydrogel microneedle patch (MNP) using gelatin methacryloyl (GelMA) as a carrier for the sustained delivery of pirfenidone (PFD), aiming to lower dosing frequency, reduce physical burden, and enhance antifibrotic efficacy. Initial in vitro studies confirmed the biocompatibility and functional performance of the Gel-PFD MNP, followed by in vivo validation in a C57 mouse model of chronic liver fibrosis. The microneedles showed complete degradation within one week, as confirmed by Cy7 fluorescence imaging. Functionally, the system suppressed fibroblast proliferation and migration in vitro. In treated mice, the Gel-PFD MNP reduced markers of fibrosis, inflammation, and apoptosis at both the transcript and protein levels, while maintaining near-normal liver enzyme values and behavioral responses. Compared to oral PFD, the microneedle-based hydrogel demonstrated superior antifibrotic activity and more consistent therapeutic outcomes. This approach holds particular promise for patients with advanced liver disease or those unable to tolerate oral medications, offering a minimally invasive and long-acting therapeutic platform [108].

Beyond drug delivery, certain hydrogels serve as biomimetic extracellular matrix scaffolds, providing structural support that facilitates hepatic regeneration and tissue repair. By modulating mechanical properties and biochemical cues within the liver microenvironment, these materials can influence hepatic stellate cell behavior, attenuate fibrogenic activation, and promote a more regenerative phenotype [109]. Although most hydrogel-based antifibrotic strategies have not been evaluated specifically in post-cholecystectomy models, they remain highly relevant to disease outcomes, as fibrosis represents a shared final pathway in NAFLD progression regardless of the initiating trigger. Integrating such approaches into the broader therapeutic framework of post-cholecystectomy NAFLD offers a rational avenue for addressing advanced disease stages and improving long-term prognosis [110].

## 8. Limitations and Future Directions

A critical limitation of the current evidence base is the absence of post-cholecystectomy-specific experimental models. Bile duct ligation combined with high-fat diet (BDL+HFD) in rodents represents the most mechanistically relevant available surrogate, recapitulating continuous bile acid flux and metabolic liver disease simultaneously, while standard models such as ob/ob mice and conventional HFD rodents inadequately reflect post-surgical bile acid kinetics, altered enterohepatic recirculation, and cholecystectomy-associated microbiome remodeling. Future studies should prioritize serum bile acid profiling (total, primary/secondary, conjugated/unconjugated fractions), fasting and postprandial FGF19 levels, and portal LPS concentrations as mechanistically relevant outcome biomarkers. Intestinal organoid and hepatocyte–Kupffer cell co-culture systems are additionally proposed as practical platforms for initial hydrogel screening, enabling bile acid exposure simulation and gut–liver axis modeling prior to in vivo validation. Long-term biocompatibility of hydrogel degradation products (chitosan oligosaccharides, alginate-derived fragments, pectin-derived galacturonic acid) remains insufficiently characterized beyond 90 days, and crosslinker residues (glutaraldehyde, genipin) require dedicated genotoxicity profiling under ICH S2(R1) guidelines. GMP scalability represents an additional challenge, as batch-to-batch variability in swelling behavior and drug loading efficiency must meet coefficient of variation targets <5% for regulatory acceptance. Oral and rectal hydrogels containing nanomaterials fall under FDA drug-device combination product guidance and EMA nanomedicine frameworks, requiring full CMC characterization as part of regulatory submission. From a clinical development perspective, phase I trials should prioritize serum bile acid profiling, postprandial FGF19 dynamics, and fecal microbiome composition as exploratory biomarkers, with adaptive phase II designs incorporating liver stiffness measurement and CAP scoring as non-invasive efficacy endpoints in the post-cholecystectomy MASLD population.

The application of hydrogel-based platforms to post-cholecystectomy NAFLD remains largely conceptual (Table 4), and future progress in this field will depend on shifting from preclinical biomaterials research toward biologically grounded, disease-specific studies. A critical priority is the development of experimental models that accurately reproduce the physiological consequences of cholecystectomy. Most currently used NAFLD models fail to capture the continuous bile flow, altered bile acid pool composition, and disrupted ileal FXR–FGF19 signaling that define the post-cholecystectomy state [111]. Surgical cholecystectomy in rodents, combined with longitudinal metabolic, microbiome, and bile acid profiling, would provide a far more relevant platform for evaluating whether hydrogel-based interventions truly modify disease mechanisms rather than merely demonstrating material performance. Incorporating endpoints such as portal endotoxin levels, intestinal permeability, hepatic triglyceride content, NAFLD activity score, and stellate cell activation markers would further align preclinical studies with clinically meaningful outcomes [5,85].

Despite their theoretical advantages, hydrogel platforms face substantial material and translational limitations that warrant acknowledgment. Synthetic hydrogels exhibit biodegradability concerns including polymer fragment accumulation and unpredictable immune responses, mechanical brittleness under intestinal peristalsis, manufacturing complexity limiting GMP scale-up, and uncertain regulatory pathways. Natural hydrogels suffer from batch-to-batch property variability, limited chemical tunability, rapid degradation causing premature drug release, potential immunogenicity from endotoxins or allergenic proteins, and mechanical weakness. Stimuli-responsive systems face specificity challenges due to inter-patient gastrointestinal variability and suboptimal response kinetics. Clinical translation is hindered by scale-up difficulties, sterilization-induced degradation, limited storage stability, substantially higher costs than conventional therapies, and absence of Phase II/III clinical data in post-cholecystectomy populations. Disease-specific challenges include uncertain optimal targeting strategies (ileum vs. colon), bile acid-mediated polymer degradation, unknown long-term efficacy, and potential drug–drug interactions. While hydrogels offer compelling advantages in spatial targeting and multifunctionality, substantial material science innovations and rigorous clinical validation are required before they represent viable alternatives to conventional therapies [112].

Future hydrogel design should also move beyond the colon-centric paradigm that currently dominates biomaterials research due to its roots in inflammatory bowel disease. In post-cholecystectomy NAFLD, the terminal ileum represents a central regulatory hub of disease biology because it governs bile acid sensing and endocrine signaling through FXR-dependent FGF19 secretion [113]. Precision-engineered hydrogels are capable of selective release in the distal ileum [114]. Achieving site-specific delivery to the terminal ileum requires precise integration of multiple design parameters. pH-responsive systems must be engineered with transition thresholds matched to the physiological pH gradient: the terminal ileum exhibits pH 7.0–7.5, compared to 6.5–6.8 in the jejunum and 5.5–6.5 in the colon. Polymer systems such as Eudragit^®^ S100 (dissolution pH ≥ 7.0), hydroxypropyl methylcellulose acetate succinate (HPMCAS, grade LG with threshold pH ~6.8–7.0), and poly(methacrylic acid-co-ethyl acrylate) copolymers with carboxyl group pKa values designed for ionization above pH 6.8 remain stable through gastric (pH 1.5–3.5) and proximal small intestinal (pH 6.0–6.5) environments, ensuring cargo protection until reaching the distal ileum where FXR–FGF19 signaling is concentrated [115]. Enzyme-responsive strategies provide complementary targeting through incorporation of azoreductase-cleavable azo bonds (–N=N–) that are selectively cleaved by bacterial azoreductases, which increase in abundance distally along the small intestine and peak in the terminal ileum and cecum [116]. Secondary enzyme-responsive linkages, such as peptide bonds cleavable by ileal brush border aminopeptidases or oligosaccharide moieties degradable by α-glucosidases, offer additional site specificity [117]. The combination of pH- and enzyme-responsive elements in dual-responsive designs enhances targeting precision through sequential triggering: pH-dependent swelling initiates in the neutral pH environment of the distal ileum, followed by enzyme-mediated hydrogel degradation and cargo release. This dual mechanism provides a fail-safe approach, as transit time variations that might cause premature exposure to colonic pH are compensated by temporal delay until sufficient bacterial enzyme activity is encountered. Mucoadhesive enhancement prolongs residence time at the ileal epithelium through thiolated polymers (e.g., chitosan-thioglycolic acid conjugates) forming disulfide bonds with cysteine-rich mucin domains, cationic polymers (chitosan, quaternized derivatives) interacting electrostatically with negatively charged mucin glycoproteins, or lectins (wheat germ agglutinin, tomato lectin) binding to specific carbohydrate epitopes on ileal enterocytes [118]. Extended contact time at the ileal epithelium maximizes FXR activation and local bile acid sequestration, compensating for the relatively short ileal transit time of 2–4 h. Finally, particle size optimization is critical: microparticle formulations should be 100–500 μm in diameter, or film-forming systems should be 10–50 μm in thickness. Particles < 10 μm risk premature absorption or M-cell uptake in Peyer’s patches (abundant in terminal ileum), while particles >1000 μm may not achieve adequate mucosal contact; intermediate sizes optimize surface area for mucoadhesion while avoiding systemic translocation [119].

Designing materials that preferentially bind hydrophobic, hepatotoxic bile acids while preserving physiological receptor activation represents a particularly promising and mechanistically sound strategy.

Equally important is the evolution of microbiota-targeted hydrogels from nonspecific probiotic carriers toward platforms informed by disease-associated microbial signatures. Predictable shifts in bile-tolerant taxa, loss of short-chain fatty acid producers, and impaired bile acid biotransformation capacity characterize post-cholecystectomy dysbiosis. Future hydrogel systems should therefore be designed to deliver defined microbial consortia with demonstrated bile acid–modifying activity, or to release tailored prebiotic substrates known to selectively enrich beneficial taxa. Responsive materials that adjust their behavior in response to microbial metabolites, such as short-chain fatty acids or redox changes, may enable dynamic interaction with the gut ecosystem rather than passive cargo release. Such approaches would move the field toward true precision microbiome engineering rather than generalized microbiota support [14,120].

The most impactful therapeutic strategies will likely be those that integrate multiple functions within a single biomaterial system. Rather than separating bile acid sequestration from drug delivery or epithelial protection, next-generation hydrogels should be designed as multifunctional constructs capable of simultaneously binding excess bile acids, reinforcing the mucosal barrier, and releasing bioactive compounds that attenuate inflammation or enhance epithelial repair. For instance, amphiphilic hydrogels that selectively sequester deoxycholic acid while gradually releasing butyrate donors, antioxidants, or epithelial growth factors could directly address the interconnected mechanisms driving gut–liver axis dysfunction. Such combinatorial designs are particularly well suited to a disease such as post-cholecystectomy NAFLD, where no single pathogenic driver operates in isolation.

At later disease stages, when fibrosis becomes the dominant determinant of prognosis, liver-targeted hydrogel strategies may offer complementary benefit. Injectable hydrogels capable of delivering antifibrotic molecules directly into hepatic tissue, including siRNA against profibrotic mediators, exosome-based therapeutics, or stem cell-derived secretomes, represent an area of substantial translational promise [108]. Moreover, emerging evidence that the mechanical properties of biomaterials can modulate hepatic stellate cell activation suggests that ECM-mimetic hydrogels with tunable stiffness may exert therapeutic effects not only through cargo delivery but also through biophysical reprogramming of the fibrotic niche. The integration of such systems with minimally invasive, image-guided delivery techniques could ultimately enable localized intervention even in advanced disease.

Similar translational principles are emerging in other biomedical fields, particularly ocular drug delivery, where hydrogels have been developed to overcome anatomical barriers such as the corneal epithelium and blood–retinal barrier that limit conventional drug bioavailability to <5%. Hydrogel-based platforms demonstrate controlled release, mucoadhesion, and improved biocompatibility, as reflected by approved products such as DEXTENZA^®^ and ReSure^®^ Sealant, while next-generation systems incorporate nanotechnology integration, 3D printing, and stimuli-responsive polymers for minimally invasive treatment of glaucoma, retinal degeneration, and dry eye disease [121].

Future studies must adopt outcome measures that reflect genuine clinical relevance rather than relying solely on material characterization. Evaluating the effects of hydrogel interventions on bile acid pool composition, circulating FGF19 levels, intestinal permeability, hepatic fat content assessed by MRI-PDFF, liver stiffness by elastography, and histological features of steatohepatitis and fibrosis will be essential for meaningful translation. Early-phase clinical trials may be most feasible in post-cholecystectomy patients with early metabolic liver changes, where modulation of the gut–liver axis has the greatest potential for disease interception rather than late-stage reversal. Taken together, the future of hydrogel-based therapy in post-cholecystectomy NAFLD will depend not on increasingly complex materials alone, but on the rational integration of biomaterials science with disease-specific biology, clinically linked endpoints, and translational feasibility.

## 9. Conclusions

Post-cholecystectomy NAFLD constitutes a distinct pathophysiological setting characterized by sustained disturbances in bile acid homeostasis, persistent remodeling of the gut microbiota, impairment of intestinal barrier integrity, and chronic activation of inflammatory and fibrogenic pathways. The convergence of these mechanisms establishes a complex gut–liver axis dysfunction that is insufficiently addressed by currently available systemic therapeutic strategies. Hydrogel-based biomaterial platforms offer a highly adaptable therapeutic approach for this context. Through their capacity for localized, stimuli-responsive, and multifunctional intervention, hydrogels enable targeted modulation of bile acid dynamics, microbial composition, mucosal integrity, and tissue-level inflammatory and fibrotic processes. The tunability of their physicochemical properties further supports the rational design of personalized therapeutic systems tailored to specific disease phenotypes. Advancing hydrogel-based strategies toward clinical translation will require coordinated interdisciplinary efforts spanning hepatology, microbiome science, biomaterials engineering, and translational medicine. Priority areas include the development of post-cholecystectomy–specific experimental models, standardized outcome measures integrating bile acid and microbiome profiling, and rigorously designed preclinical and clinical studies.

## Figures and Tables

**Table 1:** Comparison of natural and synthetic hydrogels as bile acid sequestration platforms.

Property	Natural Hydrogels	Synthetic Hydrogels
Source	Derived from biological sources (e.g., chitosan, alginate, cellulose)	Engineered from synthetic monomers (e.g., PVA, PAA, HEMA)
Biodegradability	High—readily broken down by natural enzymes or microbiota	Variable—often resistant to biodegradation
Customizability	Limited—dependent on natural polymer backbone	High—customizable via monomer choice and crosslinking
Interaction Mechanism	Primarily ionic and hydrogen bonding with bile acids	Ionic, hydrophobic, and steric interactions
Examples	Chitosan, Alginate, Pectin, Cellulose	Poly(acrylic acid), Poly(vinyl alcohol), Poly(HEMA)
Toxicity	Low toxicity; good biocompatibility	Can vary—depends on monomer used
Swelling Behavior	Moderate and pH-dependent	Highly tunable across conditions
Porosity Control	Difficult to tune precisely	Precisely adjustable by formulation

**Table 2:** Stimuli-responsive hydrogel systems for site-specific intestinal drug delivery relevant to gut–liver axis modulation.

Response Type	Hydrogel Composition	Quantitative Performance	In Vivo Efficacy	Mechanism Details	References
pH-sensitive	HA–gelatin (1:2 ratio) with curcumin-loaded chitosan microspheres	pH transition: 6.8–7.4; Release: 80% in 8 h at pH 7.4 vs. <10% at pH 1.2; Stability: >95% intact through gastric transit	IBD rat model: 65% reduction in colonic inflammation (MPO activity); 2.8-fold increase in mucosal integrity scores vs. free drug	Carboxyl ionization above pH 6.8 triggers swelling; sustained colonic curcumin release via enzymatic matrix degradation	[80]
Temperature-sensitive	Pluronic F127–HA–α-cyclodextrin with melanin–gallium nanocomplex (TMNR)	LCST: 32 °C; Gelation time: <2 min at 37 °C; G′ = 450 Pa; Retention: >48 h vs. 6 h (non-thermogelling); Mucoadhesion: 8.2× vs. control	UC mouse model: 3.2-fold TNF-α reduction; 70% decrease in DAI scores; histological recovery (inflammation score 1.2 vs. 4.8 control)	Liquid at 4 °C for injection; sol–gel transition at body temp; ROS-scavenging melanin complex; strong mucosal adhesion resists peristalsis	[81]
Enzyme-sensitive	Pectin/Ca^2+^ matrix with chitosan oligosaccharide–quercetin (COS–QT) microspheres	Target: colonic pectinase; Degradation: 90% in 6 h with enzyme vs. <5% without; Colonic delivery efficiency: 87% vs. 34% (pH-only); QT bioavailability: 4.3× improvement	Colitis model: Quercetin plasma Cmax 2.8 μg/mL (vs. 0.6 μg/mL free); 58% reduction in colonic IL-6; improved barrier function (TEER recovery 78%)	Pectin resistant to upper GI; degraded by bacterial pectinases in colon; COS enhances penetration and controlled QT release at inflammation sites	[82]
Redox-responsive	Thiolated hyaluronic acid (HASH60%, 60% thiol modification)	ROS threshold: >100 μM H_2_O_2_; Gelation: <5 min at oxidative sites; Selective adhesion: 8:1 ratio (inflamed vs. healthy mucosa); Retention: 36 h at inflamed sites	DSS-colitis mice: IL-10 increase 2.8-fold; selective accumulation in inflamed regions (fluorescence imaging); 62% reduction in oxidative stress markers (4-HNE)	Disulfide bond formation with mucin thiols under oxidative stress; ROS-triggered crosslinking; artificial mucus layer formation; protects epithelium from luminal irritants	[83]

**Table 3:** Hydrogel-Based Platforms for Localized Antifibrotic Therapy in Liver Fibrosis.

Study/System	Hydrogel Type & Delivery Mode	Therapeutic Payload	Experimental Model	Key Outcomes	Relevance to Antifibrotic Therapy
General hepatic hydrogel platforms	Injectable, liver-conforming hydrogels	Small molecules, biologics, siRNA	Preclinical liver fibrosis models	Sustained local drug release; enhanced target engagement; reduced systemic toxicity	Improves spatial control of antifibrotic therapy and minimizes off-target effects
Bolinas et al.	Injectable hydrogel, intraportal administration	Mesenchymal stem cells (MSCs)	Cirrhotic rat model	High MSC viability (89.0 ± 3.0%); sustained release (2 weeks); increased liver volume (FLR 0.57 ± 0.32) and weight (FLR 0.84 ± 0.05); reduced AST, ALT, ALP; fibrosis reduced to 4.52 ± 0.22%; higher cell retention (37.30 ± 16.10 MSCs/mm^2^)	Enhances MSC localization, viability, retention, and antifibrotic efficacy
Gu et al.	GelMA-based hydrogel microneedle patch (MNP)	Pirfenidone (PFD)	C57 mouse model of chronic liver fibrosis	Complete degradation within 1 week; suppressed fibroblast proliferation and migration; reduced fibrosis, inflammation, and apoptosis markers; near-normal liver enzymes; superior efficacy vs. oral PFD	Minimally invasive, long-acting antifibrotic delivery with improved consistency and tolerability
Biomimetic ECM hydrogels	ECM-mimicking scaffolds with tunable mechanics	None (structural/biophysical cues)	Liver regeneration and fibrosis models	Modulation of hepatic stellate cell behavior; reduced fibrogenic activation; promotion of regenerative phenotype	Supports tissue repair and fibrosis attenuation via microenvironmental regulation
Conceptual relevance to post-cholecystectomy NAFLD	Not yet directly evaluated	—	NAFLD progression models	Fibrosis identified as a shared final pathway independent of disease trigger	Provides a rational strategy for addressing advanced fibrosis in post-cholecystectomy NAFLD

**Table 4:** Critical limitations of standard NAFLD models in evaluating post-cholecystectomy hydrogel strategies.

Model Limitation	Impact on Hydrogel Validation	Translational Consequence
Bile acid dynamics	Standard models with pulsatile, meal-dependent bile secretion fail to reproduce continuous post-cholecystectomy bile flow. Hydrogel swelling behavior, binding capacity, selectivity for primary vs. secondary bile acids, and saturation kinetics cannot be accurately assessed.	Hydrogels optimized for intermittent exposure may perform suboptimally under sustained bile acid conditions in patients. Material chemistry and release kinetics based on non-physiological concentrations will not translate.
Ileal FXR–FGF19 signaling	Absence of cholecystectomy-specific disruption of ileal FXR–FGF19 feedback limits validation of ileum-targeted FXR modulators, mucoadhesive systems regulating bile acid availability, and sequestrants designed to preserve physiological signaling.	Interventions designed to restore specific signaling defects cannot be meaningfully validated in models lacking those defects. FXR agonist-loaded hydrogels may show efficacy through off-target hepatic effects rather than intended ileal mechanisms.
Microbiota composition	Post-cholecystectomy dysbiosis (bile acid-driven selection of bile-tolerant taxa, reduced secondary bile acid conversion) differs qualitatively from diet-induced dysbiosis. Probiotic strain selection for bile acid biotransformation capacity and hydrogel protective functions against bile toxicity cannot be assessed.	Probiotic formulations validated for general anti-inflammatory effects may fail to restore bile acid metabolism or colonize under high bile acid conditions. Stimuli-responsive systems targeting disease-specific metabolites will not demonstrate appropriate responsiveness.
Intestinal barrier function	Standard models do not reproduce bile acid-induced epithelial injury and barrier dysfunction. Mucoadhesive barrier-forming hydrogels, delivery systems for barrier-reinforcing agents (butyrate, growth factors), and LPS-sequestering formulations cannot be evaluated for their intended mechanisms.	Hydrogels demonstrating anti-inflammatory effects through general mechanisms may not prevent bile acid-driven epithelial damage and bacterial translocation. Portal endotoxemia patterns differ, limiting relevance of inflammatory endpoint assessment.
Material design parameters	Bile acid binding specificity, pH/enzymatic/metabolic responsiveness, site-specific targeting accuracy, and gastrointestinal transit under altered bile flow cannot be refined. Standard efficacy endpoints (triglycerides, ALT, NAS) do not capture mechanism-specific effects on FXR–FGF19 signaling, intestinal permeability, or microbial bile acid metabolism.	Fundamental material chemistry decisions (charge density, hydrophobicity, crosslinking) based on inappropriate models may compromise clinical performance. Responsive release profiles optimized for wrong environmental cues will fail in target population.
Clinical translation pathway	Absence of cholecystectomy-specific models prevents assessment of whether hydrogel interventions produce intended mechanistic effects: normalization of FXR–FGF19 signaling, restoration of secondary bile acid production, reduction in portal LPS translocation, or reinforcement of bile acid-damaged epithelium.	Promising preclinical data from standard models may not predict clinical efficacy, risking expensive trial failures. Conversely, marginally effective interventions in inappropriate models might prove highly effective in disease-relevant contexts but remain undiscovered.

This table systematically connects each experimental model limitation to specific consequences for hydrogel strategy validation and clinical translation. The absence of post-cholecystectomy-specific models affects not only efficacy assessment but fundamental material design decisions, creating substantial uncertainty in the translational pathway for these therapeutics.

## Data Availability

Not applicable.

## Abbreviations

The following abbreviations are used in this manuscript:

AFG	Snail mucus-derived glycosaminoglycan
ALP	Alkaline phosphatase
ALT	Alanine aminotransferase
AST	Aspartate aminotransferase
BA	Bile acid(s)
CC	Carboxymethyl chitosan
CUR	Curcumin
Cy7	Cyanine7 (near-infrared fluorescent dye)
DEX	Dexamethasone
DSS	Dextran sulfate sodium
ECM	Extracellular matrix
EVs	Extracellular vesicles
FXR	Farnesoid X receptor
FGF19	Fibroblast growth factor 19
F127	Pluronic F127 (poloxamer 407)
FLR	Future liver remnant
GelMA	Gelatin methacryloyl
HA	Hyaluronic acid
HA–GE	Hyaluronic acid–gelatin
HASH	Thiolated hyaluronic acid
HEMA	2-Hydroxyethyl methacrylate
HFD	High-fat diet
H_2_O_2_	Hydrogen peroxide
IBD	Inflammatory bowel disease
LPS	Lipopolysaccharide(s)
MASLD	Metabolic dysfunction-associated steatotic liver disease
MAFLD	Metabolic (dysfunction)-associated fatty liver disease
MASH	Metabolic dysfunction-associated steatohepatitis
MNP	Microneedle patch
MSCs	Mesenchymal stem cells
MRI-PDFF	Magnetic resonance imaging–proton density fat fraction
NAFLD	Non-alcoholic fatty liver disease
NASH	Non-alcoholic steatohepatitis
NF-κB	Nuclear factor kappa B
PAA	Poly(acrylic acid)
PAM	Polyacrylamide
PB	Prussian blue
PBNPs	Prussian blue nanoparticles
PFD	Pirfenidone
PVA	Poly(vinyl alcohol)
QT	Quercetin
ROS	Reactive oxygen species
SCFA	Short-chain fatty acid(s)
TGR5	Takeda G protein-coupled receptor 5
TLR4	Toll-like receptor 4
TMNR	Thermosensitive mucoadhesive nanoregulator hydrogel (as defined in the cited study)
